# Efficacy of a Web-Based Home Blood Pressure Monitoring Program in Improving Predialysis Blood Pressure Control Among Patients Undergoing Hemodialysis: Randomized Controlled Trial

**DOI:** 10.2196/53355

**Published:** 2024-08-09

**Authors:** Tingting Chen, Wenbo Zhao, Qianqian Pei, Yanru Chen, Jinmei Yin, Min Zhang, Cheng Wang, Jing Zheng

**Affiliations:** 1 School of Nursing, Guangdong Pharmaceutical University, Guangzhou, China; 2 Division of Nephrology, The Third Affiliated Hospital of Sun Yat-sen University, Guangzhou, China; 3 School of Tourism Management, Three Gorges Tourism Polytechnic College, Yichang, China; 4 Division of Nephrology, The Fifth Affiliated Hospital Sun Yat-sen University, Zhuhai, China; 5 School of Public Health, Guangdong Pharmaceutical University, Guangzhou, China

**Keywords:** hemodialysis, hypertension, home blood pressure monitoring, eHealth, randomized controlled trial

## Abstract

**Background:**

Hypertension is highly prevalent among patients undergoing hemodialysis, with a significant proportion experiencing poorly controlled blood pressure (BP). Digital BP management in this population has been underused.

**Objective:**

This study aimed to explore the efficacy of a web-based home BP monitoring (HBPM) program in improving predialysis BP control and enhancing knowledge, perception, and adherence to HBPM among patients with hypertension undergoing hemodialysis.

**Methods:**

A multicenter, open-label, randomized controlled trial was conducted at 2 hemodialysis units. Patients were randomly allocated in a 1:1 ratio to either the web-based HBPM program as the intervention group or to usual care as the control group over a 6-month period. The primary outcomes were the predialysis BP control rate, defined as less than 140/90 mm Hg, and the predialysis systolic and diastolic BP, assessed from baseline to the 6-month follow-up. Secondary outcomes included patient knowledge, perception, and adherence to HBPM, evaluated using the HBPM Knowledge Questionnaire, HBPM Perception Scale, and HBPM Adherence Scale, respectively. A generalized estimating equations analysis was used to analyze the primary outcomes in the intention-to-treat analysis.

**Results:**

Of the 165 patients enrolled in the program (n=84, 50.9% in the web-based HBPM group and n=81, 49.1% in the control group), 145 (87.9%) completed the follow-up assessment. During the follow-up period, 11 instances of hypotension occurred in 9 patients in the web-based HBPM group, compared to 15 instances in 14 patients in the control group. The predialysis BP control rate increased from 30% (25/84) to 48% (40/84) in the web-based HBPM group after the 6-month intervention, whereas in the control group, it decreased from 37% (30/81) to 25% (20/81; *χ*^2^_2_=16.82, *P*<.001; odds ratio 5.11, 95% CI 2.14-12.23, *P*<.001). The web-based HBPM group demonstrated a significant reduction after the 6-month intervention in the predialysis systolic BP (*t*_163_=2.46, *P*=.02; β=−6.09, 95 % CI −10.94 to −1.24, *P*=.01) and the predialysis diastolic BP (*t*_163_=3.20, *P*=.002; β=−4.93, 95% CI −7.93 to −1.93, *P*=.001). Scores on the HBPM Knowledge Questionnaire (*t*_163_=−9.18, *P*<.001), HBPM Perception Scale (*t*_163_=−10.65, *P*<.001), and HBPM Adherence Scale (*t*_163_=−8.04, *P*<.001) were significantly higher after 6 months of intervention.

**Conclusions:**

The implementation of a web-based HBPM program can enhance predialysis BP control and the knowledge, perception, and adherence to HBPM among patients undergoing hemodialysis. This web-based HBPM program should be promoted in appropriate clinical settings.

## Introduction

### Background

Hemodialysis has become the predominant renal replacement therapy for individuals with end-stage renal disease, the final stage of chronic kidney disease. The prevalence of patients receiving hemodialysis has surged alongside the increasing incidence of end-stage renal disease cases. Globally, the number of patients requiring renal replacement therapy is expected to reach 5.4 million by 2030, with nearly 89% of them undergoing hemodialysis [1]. According to the Chinese National Renal Data System, more than 1 million patients underwent dialysis in 2022 [2].

Hypertension is nearly ubiquitous among patients receiving hemodialysis, who often have suboptimal blood pressure (BP) control. The epidemiology of hypertension in this population varies across studies, depending on the definition of hypertension and the method used to measure BP. The overall prevalence of hypertension in patients undergoing hemodialysis ranged from 80% to 90% [3-5]. The European Registry of Cardiovascular and Renal Medicine of the European Renal Association—European Dialysis and Transplant Association showed that hypertension, defined as having ambulatory BP ≥130/80 mm Hg at 48 hours or current use of medications to reduce BP, was prevalent in 84.3% of patients undergoing hemodialysis [3]. Another study based on the criteria of predialysis BP ≥140/90 mm Hg or current use of antihypertensives indicated that 86.2% of patients undergoing hemodialysis have hypertension [4]. Additionally, the China Dialysis Outcomes and Practice Patterns Study reported that 87.3% of patients undergoing hemodialysis were complicated by hypertension [5]. Although 86.8% of these patients received pharmacological treatment, fewer than 30% had their BP well controlled [3].

Increased BP in patients undergoing hemodialysis has been found to have a linear association with adverse cardiovascular events and mortality. Recent studies have identified a U-shaped or J-shaped correlation between BP before or after dialysis and the risk of all-cause mortality in patients undergoing hemodialysis. This phenomenon, termed the “reverse epidemiology” of hypertension, is predominantly observed within dialysis units [6,7]. Elevated BP levels measured outside the dialysis unit demonstrated better prognostic capacity for adverse cardiovascular events and mortality compared to measurements taken within the dialysis unit [8-10]. Therefore, it is imperative to develop a more effective BP management model for patients undergoing hemodialysis, especially BP measurements taken outside the dialysis unit, to optimize the reduction in mortality risk.

BP monitoring plays a crucial role in the management of BP, representing a fundamental prerequisite therein, and is a crucial tool for the identification, diagnosis, and prognostic evaluation of hypertension. For patients undergoing hemodialysis, BP measurements are taken at the hemodialysis unit and outside the hemodialysis unit, and the latter consists of home BP monitoring (HBPM) and ambulatory BP monitoring [11]. Despite the conventional clinical application of BP measurements taken within dialysis units for diagnosing hypertension, the limitations of this approach persist [11]. In contrast, HBPM offers improved reproducibility [12], superior diagnostic accuracy [13], a more intimate nexus with target organ damage [14], and more prognostic information [15] and serves as a guide for long-term antihypertensive regimens [15]. Compared to ambulatory BP monitoring, HBPM has advantages in terms of lower cost, simpler operation, and greater patient acceptance [11]. Furthermore, the existing literature highlighted that positive behavior with HBPM can promote patient adherence to obtaining or reporting home BP, achieving better management of BP [16].

Under the proposal of national information health care policies [17,18], the maturation of information technology, and the amalgamation of network information, medical health services have engendered novel perceptions for the management of BP. Within the realm of existing digital interventions for BP, a variety of modalities such as BP telemonitoring, smartphone app-based tracking, phone calls, websites, emails, and SMS text messages have been used, demonstrating notable efficacious results [19]. Compared to conventional BP management paradigms, digital BP management is more convenient and flexible, with greater patient acceptance [19]. However, digital BP management has been applied predominantly in the primary hypertension population, rather than in patients undergoing hemodialysis [19]. It is unclear whether the efficacy of digital BP management modalities has the potential to influence predialysis BP control in patients with hypertension undergoing hemodialysis.

### Objective

To bridge this knowledge gap, our goal was to develop a customized digital BP management program for patients with hypertension undergoing hemodialysis and to evaluate its long-term feasibility, efficacy, and safety in these patients. A 6-month, prospective, multicenter, randomized controlled trial was designed, with the aim of a comprehensive investigation of the efficacy of a digital BP management program in improving predialysis BP control rates, along with an evaluation of its implications for cognition, perception, and adherence to HBPM among patients with hypertension undergoing hemodialysis.

## Methods

### Study Design

This study was a multicenter, open-label, randomized controlled trial comparing the web-based HBPM program against usual care for patients with hypertension undergoing hemodialysis, which was performed at the dialysis centers of 2 tertiary hospitals in Guangdong Province, China, from August 2022 to February 2023. The design of the study was in accordance with the specifications outlined in the CONSORT-eHEALTH checklist (Checklist 1).

### Ethical Considerations

The study protocol was registered with the Chinese Clinical Trial Registry (ChiCTR2100051535) and was approved by the ethics committees of Zhongshan Third Affiliated Hospital of Sun Yat-sen University ([2019] 02-520-01) and Zhongshan Fifth Affiliated Hospital of Sun Yat-sen University ([2022] K141-1). The authors adhered to the tenets of the Declaration of Helsinki and its amendments. Written informed consent was obtained from each participant. All data collected from participants were recorded in an anonymized format. Participants in both groups were paid equally, such as a sphygmomanometer or an equivalent gift. These details were conveyed to the participants at the time of signing the informed consent form.

### Participants

The inclusion criteria were (1) aged between 18 to 80 years; (2) mean predialysis systolic BP (SBP) of ≥140 mm Hg, mean diastolic BP (DBP) of ≥90 mm Hg over 3 consecutive sessions, the use of antihypertensive medication [20], or any combination of the above; (3) stage 5 and estimated glomerular filtration rate less than 15 mL/(min×1.73 m^2^); (4) dialysis initiation period exceeding 3 months; (5) competencies in communication, perception, and learning; and (6) proficiency in smartphone operation and willingness to participate in the study. Exclusion criteria included patients who (1) transferred to intensive care units for any reason; (2) experienced complicating infection or bleeding; (3) died during the research period; (4) had mental or physical disabilities impairing their ability to respond to inquiries; (5) were unable to see or hear or were unable to measure BP due to upper-limb disability; or (6) demonstrated an inability to engage in self-care, as indicated by a score below 60 on the Activities of Daily Living scale. Participants were enrolled by researchers (YC and JY).

A simple randomization procedure was performed by researchers (JZ and MZ) using a random number table generated from the list of patients undergoing hemodialysis, at a 1:1 randomization ratio. Since patient dialysis management was conducted by different groups, each group of 8‐10 patients had a relatively fixed dialysis time, dialysis area, and group of attending nurses. To minimize bias, the allocation of participants into study groups was conducted using a block randomization procedure. The random allocation sequence was generated according to the dialysis group through a publicly available web-based tool [21].

### Intervention

#### Intervention Group

##### Development of the Web-Based HBPM Program

Concomitant with the management of the disease according to the *Chinese Standard Operating Procedures for Blood Purification* [22], the patients in the intervention group received complementary management of BP in the form of the web-based HBPM program. The theoretical framework of this program was developed according to the Health Promotion Model [23]. The program protocol was methodically formulated by the research group by systematically searching, screening, evaluating, and synthesizing the literature and based on clinical experience. Subsequently, the final version of the web-based HBPM program was validated using a peer-reviewed expert consensus approach.

##### Web-Based HBPM Program Protocol

**Figure 1.**
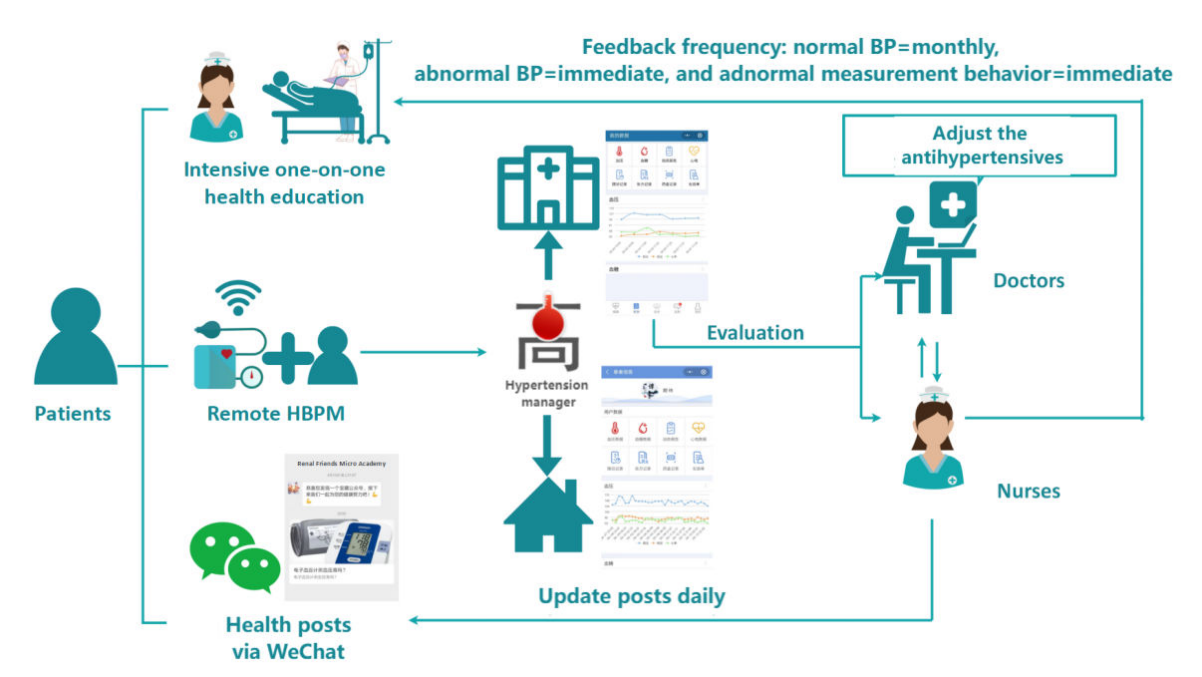
The framework of the web-based HBPM program. BP: blood pressure; HBPM: home blood pressure monitoring.

The web-based HBPM program was characterized by a tripartite framework that encompassed intensive one-on-one health education; remote HBPM; and health posts disseminated through the WeChat platform (Figure 1). The program was implemented by health care professionals, including doctors and nurses in the hemodialysis centers, who received training in hypertension management conducted by the research group prior to the intervention.

##### Intensive Individualized Health Education

Intensive, one-on-one health education was a component of the web-based HBPM program. At the beginning of the intervention, the nurses in charge of the patients in the web-based HBPM group implemented dedicated, intensive, and individualized health education according to the *Nurse Education Manual*, which was formulated through a review of the literature. First, nurse leaders provided comprehensive instruction to participants that included the proper techniques for normative BP measurement, including the required measurement frequency, time, and recording of BP readings. The instruction was provided in half an hour. Subsequently, nurse leaders connected the patient’s sphygmomanometers to the WeChat platform to facilitate remote HBPM. After that, nurse leaders provided monthly one-on-one education for 6 months. Nurse leaders offered feedback according to patients’ home BP readings from the previous month; laboratory test results; and assessments of patient knowledge, perceptions, and adherence to HBPM. They provided personalized coaching to patients monthly, using positive encouragement to improve patients’ confidence in monitoring their BP at home appropriately. Furthermore, if patients developed conditions (abnormal BP, abnormal measurement behavior, and relative interdialytic weight gain ≥5%), immediate education was required. Two researchers (TC and WZ) were responsible for providing web-based counseling and responding to patients at any time.

##### Remote HBPM

The core component of the web-based HBPM program was remote HBPM. To establish a digital conduit for the transmission and recording of BP data, internet-connected sphygmomanometers were used. These devices automatically uploaded BP measurement data to the cloud for monitoring purposes. The cloud was divided into 2 portals: the health care professional portal and the patient portal. Both health care professionals and patients used this cloud on the internet to collaboratively monitor patients’ home BP.

After registering the sphygmomanometers, which automatically transmitted BP measurement data to the patient’s WeChat account, nurse leaders guided patients to check BP reports on the web. The patients were then instructed to monitor their home BP with these sphygmomanometers following the nurse’s instructions. The requirements for HBPM behavior were as follows: for patients whose BP met the standards (home BP <135/85 mm Hg [24]), it was recommended that they monitor their BP 1-2 days per week; for patients with unstable or substandard BP, it was recommended that they measure their BP 5-7 days per week. During these monitoring sessions, measurements were performed both in the morning and in the evening, following a rest period of 1-2 minutes.

Nurse leaders tracked both home BP and dialysis BP using the health care professional portal via the cloud and hemodialysis machine. Nurse leaders observed and evaluated the measurements of BP monthly through the health care professional portal and hemodialysis machine. They observed abnormal BP instances, including uncontrolled home BP (defined as an average home BP of ≥135/85 mm Hg [24]), interdialytic hypotension (defined as a reduction in SBP of 20 mm Hg or more or a decrease in mean arterial pressure by 10 mm Hg, accompanied by symptoms on nondialysis days), intradialytic hypotension (a decrease in BP accompanied by symptoms in dialysis procedure [25]), and uncontrolled predialysis BP (an average predialysis BP of ≥140/90 mm Hg [22]). They provided feedback to physicians about abnormal BP and discussed the adjustments to achieve BP management. Meanwhile, nurse leaders evaluated patient compliance with HBPM through the health care professional portal.

The physicians used the health care professional portal on the cloud and hemodialysis machine to monitor the patients. Initially, they adopted comprehensive nonpharmacological strategies, such as adjusting patient’s dry weight and controlling fluid and sodium intake. If these measures did not normalize the patient’s BP (predialysis BP <140/90 mm Hg), the patient’s antihypertensive regimen would be adjusted. Additionally, adjustments to ultrafiltration and dry weight would be made, and instructions on managing interdialytic hypotension would be conducted according to the *China Standard Operating Procedures for Blood Purification* [22] to ensure safety at home. Throughout the continuum of BP management, health care professionals remained concerned about the effect of BP reduction, patient feelings, and adverse events through BP management and set goals for the next stage.

##### Health Posts via WeChat

Health posts via WeChat served as an integral component of the web-based HBPM program. Before the intervention, nurse leaders assisted patients in subscribing to a public WeChat account dedicated to self-care knowledge and skills related to BP management for patients undergoing hemodialysis, created by the researchers in this study. A researcher updated the health posts on this public WeChat account and distributed them to patients daily, while also monitoring their engagement and encouraging active participation. The WeChat health posts covered 35 topics consisting of 196 posts, including discussions on HBPM, fluid management, and calcium-phosphorus management. The content was regularly updated to ensure relevance and timeliness.

### Control Group

Participants assigned to the control group received routine disease management based on the *China Standard Operating Procedures for Blood Purification* [22]. Patients were advised to measure their home BP, record the results in a notebook, and return it to the health care professional voluntarily 1 or several months later. If the home BP of the patients did not reach the standard BP, the physicians would adjust treatment through nonpharmacological or pharmacological methods to control the BP of the patients according to *Chinese Standard Operating Procedures for Blood Purification* [22]. Patients were provided with education from nurses on BP self-monitoring and BP management at the initiation of antihypertensive therapy.

### Outcomes and Instruments

The primary outcomes were the predialysis BP control rate, with targets set at a predialysis BP of less than 140/90 mm Hg [22], and the predialysis SBP and DBP. Secondary outcomes included patient knowledge, perception, and adherence to HBPM.

#### The Predialysis BP and HBPM Instrument

Predialysis BP was obtained before dialysis treatment using the automated BP monitor attached to the hemodialysis machine (Dialog+) in the dialysis unit. Predialysis BP was measured using standardized in-office methods [11]. At baseline and at each follow-up (after 1, 3, and 6 months of the study period), the BP readings were collected and averaged from the latest week, measured by the automated BP monitor attached to the hemodialysis machine. The outcome assessors (QP and CW) for BP were blinded to the intervention assignment.

Home BP was measured by an automatic electronic sphygmomanometer (A666G; Kangkang Shengshi Information Technology Co., Ltd [26]) in the web-based HBPM group.

#### HBPM Knowledge Questionnaire

Cognition of hypertension prevention and BP monitoring was measured using the HBPM Knowledge Questionnaire (HBPMKQ), which was developed according to the guidelines [27,28] by the research group. It consists of 20 multiple-choice questions with a potential total score ranging from 0 to 60, with lower scores indicating weaker knowledge. The difficulty coefficient and discrimination indices of the questionnaire were moderate and well defined [29].

#### HBPM Perception Scale

The perception of HBPM was assessed using the HBPM Perception Scale (HBPMPS) [29]. This scale is a 5-point Likert scale comprising 27 items across 5 dimensions: perceived benefit of HBPM, perceived barriers to HBPM, perceived self-efficacy of HBPM, situational influence, commitment to an HBPM plan. The total score ranges from 27 to 135, with lower scores indicating a worse attitude toward HBPM.

#### HBPM Adherence Scale

Adherence to HBPM was measured using the HBPM Adherence Scale (HBPMAS) [30]. The HBPMAS uses a 5-point Likert scale and consists of 8 items, with lower scores indicating poorer adherence to HBPM.

### Data Collection

A trained investigator collected the data by interviewing patients. Sociodemographic and clinical characteristics were collected by a trained investigator using electronic questionnaires and by retrieving clinical data from the charts at the beginning of the study. The predialysis BP values between the 2 groups were recorded by a trained investigator and measured during the same period at baseline (T0) and at the 1-, 3-, and 6-month follow-ups (T1, T2, and T3, respectively). Electronic questionnaires for the HBPMKQ, HBPMPS, and HBPMAS were completed by both groups at the beginning of the study and at the 1-, 3-, and 6-month follow-ups.

### Statistical Analysis

To test the differences between the 2 groups, α was set at .05 and the test power (1 – β) was set at .90. A previous study reported a predialysis BP control rate of 28.2% (defined as <140/90 mm Hg) [3]. Assuming a 25% improvement after the intervention, the sample size was calculated to be 124 cases according to the 2 independent sample rates. Considering a dropout rate of 20%, at least 155 patients must be enrolled.

All analyses used intention-to-treat principles, and missing data were input using the last-observation-carried-forward approach. Sociodemographic and clinical characteristics and study outcomes were described as mean (SD) or n (%). The comparison of sociodemographic and clinical variables and study outcomes between the 2 groups was conducted using independent-sample (2-tailed) *t* tests and *χ*^2^ tests. The primary outcomes were analyzed using generalized estimating equations. Model 1 was adjusted for group and time. Model 2 also controlled for hospital, age, sex, education, employment status, and marital status. Model 3 additionally controlled for smoking, BMI, the number of antihypertensive agents, duration of dialysis, interdialytic weight gain, urea clearance index, and weekly dialysis frequency. All analyses were processed in SPSS software (version 25.0; IBM Corp).

## Results

### Enrollment and Participant Allocation

**Figure 2.**
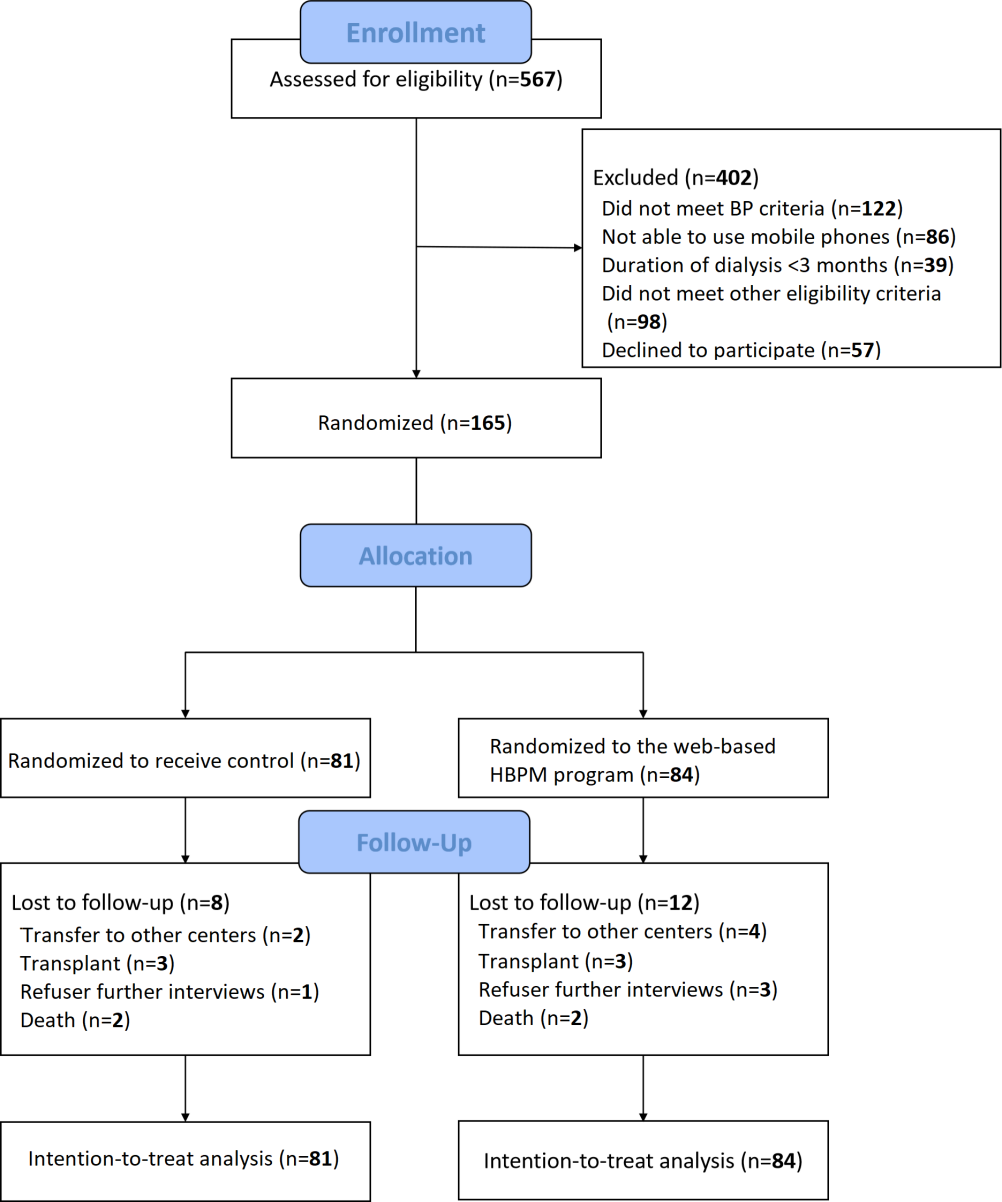
Flow diagram of the web-based HBPM program. BP: blood pressure; HBPM: home blood pressure monitoring.

During the recruitment periods, a total of 567 patients undergoing hemodialysis were assessed for eligibility. Among them, 165 enrolled patients were randomly assigned to either the web-based HBPM program group (n=84, 50.9%) or the usual care group (n=81, 49.1%; Figure 2). Dialysis unit A comprised 81 (49.1%) patients, whereas dialysis unit B comprised 84 (50.9%) patients. No statistically significant differences were observed in the demographic and clinical characteristics of the participants between the 2 dialysis units (all *P*>.05). A total of 20 participants prematurely discontinued the study, primarily due to transferring to other dialysis centers, receiving kidney transplantation, declining further interviews, and death. Throughout the follow-up period, the web-based HBPM program group reported 11 instances of hypotension in 9 patients, including 10 instances of intradialytic hypotension and 1 instance of interdialytic hypotension. The control group reported 15 instances of hypotension in 14 patients, including 11 instances of intradialytic hypotension and 4 instances of interdialytic hypotension. No hypotension-related adverse events, such as cardiovascular events (eg, acute myocardial infarction or ischemic stroke) or falls, were observed in either group.

### Demographic and Clinical Characteristics of the Participants

Of the 165 patients, the mean age was 53.7 (14.0) years and 66.1% (n=109) were male. The mean duration of hemodialysis was 57.8 (45.9) months. Glomerulonephritis (n=70, 42.4%) was the main etiology of renal disease. A total of 84.8% (n=140) of patients received treatment for their hypertension, 43.6% (61/140) were treated with 1 antihypertensive agent, and 56.4% (79/140) were given a combination of antihypertensives. Among those who received a combination of antihypertensives, the combination of calcium channel blockers+β-blockers+angiotensin II receptor antagonists was the most common (25/79, 32%), followed by calcium channel blockers+β-blockers (22/79, 28%). Except for education, other characteristics were similar between the 2 groups (Table 1).

**Table 1.** Demographic and clinical characteristics of participants (n=165).

Demographic and clinical characteristics			Web-based HBPM^a^ group (n=84)	Control group (n=81)	*t* test or chi-square (*df*)	*P* value
**Demographic information**						
	Age (years), mean (SD)		53.15 (14.65)	54.17 (13.36)	.47 (163)	.64
	**Sex, n (%)**				.03 (1)	.87
		Male	56 (67)	53 (65)		
		Female	28 (33)	28 (35)		
	**Education, n (%)**				7.98 (3)	.046
		Elementary school	8 (10)	13 (16)		
		Junior middle school	21 (25)	32 (40)		
		High school	26 (31)	14 (17)		
		College or above	29 (34)	22 (27)		
	**Employment status, n (%)**				2.00 (2)	.37
		Employed	27 (32)	20 (25)		
		Unemployed	32 (38)	29 (36)		
		Retired	25 (30)	32 (40)		
	**Marital status, n (%)**				.25 (1)	.62
		Married or cohabiting	66 (79)	61 (75)		
		Single, divorced, or widowed	18 (21)	20 (25)		
	**Current smoking, n (%)**				.26 (1)	.61
		Yes	15 (18)	17 (21)		
		No	69 (82)	64 (79)		
**Medical history**						
	**Cause of the renal disease, n (%)**				1.02 (5)	.96
		Glomerulonephritis	34 (40)	36 (44)		
		Diabetic nephropathy	20 (24)	21 (26)		
		Hypertensive nephropathy	10 (12)	9 (11)		
		Polycystic renal disease	5 (6)	3 (4)		
		Obstructive nephropathy	3 (4)	3 (4)		
		Other or unknown	12 (14)	9 (11)		
	**Comorbidities, n (%)**					
		Diabetes mellitus	23 (27)	26 (32)	.44 (1)	.51
		Heart failure	8 (10)	9 (11)	.03 (1)	.86
		Peripheral vascular disease	10 (12)	14 (17)	.96 (1)	.33
		Cerebrovascular disease	7 (8)	6 (7)	.05 (1)	.83
		Asthma or COPD^b^	2 (2)	1 (1)	.30 (1)	.58
		Cancer	4 (5)	4 (5)	.003 (1)	.96
	BMI (kg/m^2^), mean (SD)		21.08 (3.46)	22.55 (3.36)	.88 (163)	.38
	**Family history of hypertension, n (%)**				.84 (1)	.36
		Yes	37 (44)	30 (37)		
		None	47 (56)	51 (63)		
	**Number of antihypertensive agents used, n (%)**				4.65 (3)	.20
		0	8 (10)	17 (21)		
		1	35 (42)	26 (32)		
		2	21 (25)	20 (25)		
		≥3	20 (24)	18 (22)		
	Duration of dialysis (month), mean (SD)		54.62 (47.91)	61.07 (43.78)	−.90 (163)	.37
	IDWG/d^c^, mean (SD)		.83 (.38)	.87 (.44)	.72 (163)	.47
	**Weekly dialysis frequency, n (%)**				.03 (1)	.86
		2	9 (11)	8 (10)		
		3	75 (89)	73 (90)		
	Kt/V^d^, mean (SD)		1.36 (0.27)	1.40 (0.33)	.90 (163)	.37

a HBPM: home blood pressure monitoring.

b COPD: chronic obstructive pulmonary disease.

c IDWG/d: interdialytic weight gain.

d Kt/V: urea clearance index, calculated as the ratio of the urea clearance by the dialyzer and the volume for a particular dialysis duration.

### Predialysis BP Control Rate, BP, Knowledge of Patients, Perception, and Adherence to the HBPM

Descriptive statistics were used to describe the control rate of predialysis BP; predialysis BP; and HBPMKQ, HBPMPS, and HBPMAS scores at baseline and at the 1-, 3-, and 6-month follow-ups (Table 2). Preliminary calculations suggested that the web-based HBPM program improved patient’ predialysis BP control rate; predialysis BP; and the patients’ knowledge, perception, and adherence to the HBPM after the 6-month intervention.

**Table 2.** Descriptive statistics for outcomes from baseline to the 6-month postintervention follow-up.

Variables and time point		Web-based HBPM^a^ group (n=84)	Δ (T3 – T0)	Control group (n=81)	Δ (T3 – T0)	*t* test or chi-square (*df*)	*P* value
**Predialysis BP^b^ control, n (%)**			15 (18)		−10 (−12)	16.82 (2)	<.001
	T0^c^	25 (30)		30 (37)			
	T1^d^	32 (38)		22 (27)			
	T2^e^	30 (36)		21 (26)			
	T3^f^	40 (48)		20 (25)			
**Predialysis SBP^g^ (mm Hg), mean (SD)**			−3.93 (13.16)		2.16 (18.32)	2.46 (163)	.02
	T0	146.75 (15.97)		144.05 (17.70)			
	T1	146.94 (16.93)		145.69 (17.80)			
	T2	145.85 (15.10)		146.33 (15.83)			
	T3	142.82 (15.45)		146.21 (16.56)			
**Predialysis DBP^h^ (mm Hg), mean (SD)**			−2.16 (10.40)		2.77 (9.38)	3.20 (163)	.002
	T0	83.71 (12.19)		82.02 (11.31)			
	T1	84.01 (11.68)		83.38 (12.89)			
	T2	82.87 (11.96)		83.86 (12.10)			
	T3	81.55 (12.20)		84.79 (12.80)			
**HBPMKQ^i^ score, mean (SD)**			16.5 (9.96)		2.07 (10.24)	−9.18 (163)	<.001
	T0	40.36 (9.46)		41 (7.79)			
	T1	48.71 (6.22)		42.67 (7.75)			
	T2	53.71 (4.94)		44.33 (7.75)			
	T3	56.86 (5.72)		43.07 (8.63)			
**HBPMPS^j^ score, mean (SD)**			18.32 (15.76)		−4.19 (11.04)	−10.65 (163)	<.001
	T0	95.7 (9.44)		91.09 (7.26)			
	T1	102.12 (9.26)		96.8 (10.82)			
	T2	103.19 (7.57)		95.72 (9.61)			
	T3	114.02 (10.58)		86.9 (9.32)			
**HBPMAS^k^ score, mean (SD)**			6.44 (5.97)		−2.43 (8.08)	−8.04 (163)	<.001
	T0	31.18 (4.70)		29.86 (6.06)			
	T1	36.46 (3.65)		30.02 (6.82)			
	T2	35.06 (3.38)		28.38 (7.41)			
	T3	37.62 (3.65)		27.43 (7.51)			

a HBPM: home blood pressure monitoring.

b BP: blood pressure.

c T0: at the baseline of the study.

d T1: 1-month follow-up.

e T2: 3-month follow-up.

f T3: 6-month follow-up.

g SBP: systolic blood pressure.

h DBP: diastolic blood pressure.

i HBPMKQ: Home Blood Pressure Monitoring Knowledge Questionnaire.

j HBPMPS: Home Blood Pressure Monitoring Perception Scale.

k HBPMAS: Home Blood Pressure Monitoring Adherence Scale.

### Effect of the Web-Based HBPM Program on Predialysis BP

The results of the generalized estimating equation analysis demonstrated the importance of the duration of follow-up and intergroup interaction across various models (Table 3 and Multimedia Appendix 1). In model 1, controlling for groups and time effects, a significant difference was found between the web-based HBPM group and the control group regarding the predialysis BP control rate at T1 (odds ratio [OR] 2.29, 95% CI 1.16‐4.53; *P*=.02), T2 (OR 2.20, 95% CI 1.19‐4.08; *P*=.01), and T3 (OR 3.85, 95% CI 1.89‐7.83; *P*<.001). The OR of the predialysis BP control rate increased slightly after an additional adjustment for the demographic data of the patients in model 2, while after further adjustment for the clinical characteristics of the patients in model 3, the odds markedly increased. The 2 groups were affected differently during the follow-up period. The 3 models indicated a significant reduction in both predialysis SBP and DBP after 6 months of intervention (Multimedia Appendix 2).

**Table 3.** Analysis of the general estimating equations for the effect of the intervention on predialysis BP^a^ control rate (n=165). Model 1 controlled for group and time; model 2 also controlled for hospital, age, sex, education, employment status, and marital status; and model 3 additionally controlled for smoking, BMI, the number of antihypertensive agents, duration of dialysis, interdialytic weight gain, urea clearance index, and frequency of weekly dialysis.

Outcomes			Model 1, OR^b^ (95% CI)	*P* value	Model 2, OR (95% CI)	*P* value	Model 3, OR (95% CI)	*P* value
**Predialysis BP control rate**								
	Group^c^		0.72 (0.38-1.38)	.32	1.55 (0.78-3.07)	.21	0.70 (0.33-1.48)	.35
	**Time** ^**d**^							
		1 month (T1)	0.63 (0.40-1.01)	.06	1.64 (0.99-2.71)	.06	0.57 (0.32-1.02)	.049
		3 months (T2)	0.60 (0.37-0.97)	.04	1.75 (1.04-2.95)	.04	0.53 (0.29-0.96)	.04
		6 months (T3)	0.56 (0.31-0.99)	.048	1.876 (1.00-3.51)	.049	0.49 (0.24-1.00)	.049
	**Group** ^**c**^ **×time** ^**d**^							
		1 month	2.29 (1.16-4.53)	.02	2.43 (1.17-5.06)	.02	2.74 (1.19-6.32)	.02
		3 months	2.20 (1.19-4.08)	.01	2.33 (1.20-4.51)	.01	2.62 (1.23-5.59)	.01
		6 months	3.85 (1.89-7.83)	<.001	4.22 (1.96-9.09)	<.001	5.11 (2.14-12.23)	<.001

a BP: blood pressure.

b OR: odds ratio.

c Reference: baseline.

d Reference: control group.

## Discussion

### Principal Findings

To our knowledge, this is the first study to develop a digital BP management program specifically tailored for patients with hypertension undergoing hemodialysis. Additionally, it explores the application of a web-based HBPM program to manage BP outside of the hemodialysis unit in this patient population. Unlike previous studies that have focused on the management of BP in patients undergoing hemodialysis by adopting conventional HBPM [12,15,16,31]; modifications to hemodialysis regimens [32-34]; patient education and counseling intervention [35,36]; reduction in dry weight [37,38]; dietary sodium restriction [37]; physical exercise [39]; continuity of care through web-based education, telephone visits, and outpatient visits [40]; and cognitive behavior therapy [41], our study innovatively used remote HBPM. Sheppard et al [42] highlighted the categorization of HBPM interventions into 4 intensity levels for managing hypertension. They emphasized that a multicomponent approach integrating goal setting, health education, telemonitoring, and prompt feedback from health care professionals could achieve better BP control. The web-based HBPM program is a high-intensity, level-4 intervention for people with hypertension undergoing hemodialysis, which was complemented by 2 educational approaches: health posts via WeChat and intensive personalized health education. In this study, a total of 165 patients with hypertension undergoing hemodialysis were initially included, with 145 patients ultimately completing the study. The results unequivocally demonstrated the beneficial impact of the web-based HBPM program in patients undergoing hemodialysis. The program led to enhancements in predialysis BP control rates; predialysis BP; and patient knowledge, perception, and adherence to HBPM, which were particularly evident at the 6-month follow-up assessment. These findings support the viability, efficacy, and security of implementing the web-based HBPM program in patients undergoing maintenance hemodialysis.

In this study, preliminary analysis suggested a notable improvement in the predialysis BP control rate among patients who participated in the web-based HBPM program. Specifically, the proportion of the patients achieving predialysis BP control in the web-based HBPM group increased from 30% (25/84) to 48% (40/84), whereas in the control group, it decreased from 37% (30/81) to 25% (20/81; *P*<.001). Similar results were also observed in patients with essential hypertension [43], revealing an 11.6% improvement in the BP control rate after a 12-week HBPM intervention. The initial predialysis BP control rate among all participants in our study was 33.3% (55/165), which is consistent with the study by Sarafidis et al [3], which reported a rate of 28.2% among European patients undergoing hemodialysis. The implementation of a web-based HBPM program resulted in a remarkable elevation in the predialysis BP control rate. The elevated rate was assumed to be a consequence of improved adherence to treatment, resulting in better control of predialysis BP. The OR of the control rate for BP before dialysis increased when further controlling for clinical characteristics, compared to controlling for demographic characteristics alone. The multivariate adjustment models demonstrated that the predialysis BP control rate in the web-based HBPM group was 2.74 times that of the control group at the 1-month intervention time point, 2.62 times at the 3-month intervention time point, and 5.11 times at the 6-month follow-up. This indicated that the predialysis BP control rate was also affected by clinical characteristics, especially dry weight, ultrafiltration, dialysis adequacy, and weekly dialysis frequency. The intricate interaction between BP and volume management is well recognized in patients undergoing hemodialysis [44]. By controlling for clinical characteristics, the web-based HBPM program has been found to more effectively promote the predialysis BP control rate.

The results showed a significant reduction in predialysis SBP and DBP in the web-based HBPM group compared to the control group at the 6-month follow-up (SBP: β=−6.09, 95% CI −10.94 to −1.24; DBP: β=−4.93, 95% CI −7.93 to −1.93; Multimedia Appendix 2), despite no significant reduction in BP at the 1-month and 3-month follow-ups (*P*>.05). These findings aligned with a previous study, which found that guiding antihypertensive therapy based on BP measurements at home and before dialysis exhibited a significant 6‐ to 10–mm Hg change in BP compared to those subjected only to predialysis BP measurements [15]. Our findings contribute substantially to the literature on the utility of HBPM in reducing BP among patients undergoing hemodialysis. Despite these promising findings, the development of HBPM to treat patients with hypertension undergoing hemodialysis is limited [11,45]. Adoption rates among these patients are low [11], with only 2% adhering to standardized BP measurement protocols and 61.1% measuring their BP independently at home [45]. Therefore, it is imperative to advocate for the widespread implementation of standardized HBPM in patients undergoing hemodialysis to improve hypertension management. Information technology under favorable conditions could promote efficient management of BP among patients.

In this study, to systematically investigate knowledge, perception, and adherence to HBPM, improvements in secondary outcomes were observed after a 6-month intervention (Δ_HBPMKQ_=14.4, SD 8.3; Δ_HBPMPS_=22.5, SD 9.4; Δ_HBPMAS_=8.9, SD 6.0), which was consistent with primary hypertension research [46]. In a study by Sun et al [46], a remote interactive approach “Internet+” was used to promote the management of BP, leading to a 50.25% increase in the proportion of timely measurements and a 45.18% increase in high-degree disease awareness after the intervention. In the short term, the web-based HBPM program used health education to improve the patient’s knowledge and improved perception of the benefits, barriers, and self-efficacy associated with HBPM. This, in turn, facilitated the adoption of health-orientated behaviors by patients. In essence, the knowledge, perception, and behavior of the patients about HBPM exhibited a rapid increase and eventually stabilized over a period of time. However, translating these enhanced perceptions and behaviors into favorable health outcomes presented a certain temporal delay, indicating an existing delay. Thus, the introduction of the clinically relevant health management model should consider the overall participation of individuals, as well as the impact on cognition, perception, and behavior of the corresponding health management, to promote the improvement of outcomes.

### Limitations

In general, there were several limitations in this trial. First, due to the nature of the health behavior intervention, participant blinding was not feasible. Similarly, health care professionals could not be blinded as the same individuals enrolled participants and conducted follow-ups. Second, due to resource constraints, the study relied on a relatively short follow-up period. Lastly, given the modest sample size, the web-based HBPM program appeared to have improved BP control. However, its impact on health outcomes, such as cardiovascular events or the enhancement of dialysis services, remained uncertain. This underscores the necessity for further research with larger sample sizes across multiple centers to verify the web-based HBPM program’s clinical applicability and reliability in future studies.

### Conclusions

The web-based HBPM program introduced in this study produced significant improvements in the predialysis BP control rate and the knowledge, perception, and adherence to HBPM by patients with hypertension undergoing hemodialysis. BP was only significantly decreased at the 6-month follow-up. The web-based HBPM program exhibited benefits in BP control, potentially stemming from improved treatment adherence and optimized prescriptions. This study advocates for the implementation of the web-based HBPM program within the framework of a hemodialysis unit, as aligned with the prevailing circumstances.

## Supplementary material

10.2196/53355 Multimedia Appendix 1 Forest plots of the predialysis blood pressure control rate in the interaction of intergroup and research time based on the 3 tested models.

10.2196/53355 Multimedia Appendix 2 Analysis of the general estimating equations for the effect of the intervention on predialysis blood pressure (n=165).

10.2196/53355 Checklist 1 CONSORT-eHEALTH checklist.

## Abbreviations

BP: blood pressure
DBP: diastolic blood pressure
HBPM: home blood pressure monitoring
HBPMAS: Home Blood Pressure Monitoring Adherence Scale
HBPMKQ: Home Blood Pressure Monitoring Knowledge Questionnaire
HBPMPS: Home Blood Pressure Monitoring Perception Scale
OR: odds ratio
SBP: systolic blood pressure
