# Supplementary material for: Efficacy of a Web-Based Home Blood Pressure Monitoring Program in Improving Predialysis Blood Pressure Control Among Patients Undergoing Hemodialysis: Randomized Controlled Trial
Source: JMIR Mhealth Uhealth. 2024 Aug 9;12:e53355. doi: 10.2196/53355 (PMC11350391; doi:10.2196/53355)
Supplement: Multimedia Appendix 1 [file mhealth-v12-e53355-s001.docx]

**Multimedia Appendix 1.** Forest plots of the predialysis blood pressure control rate in the interaction of intergroup and research time based on the three tested models


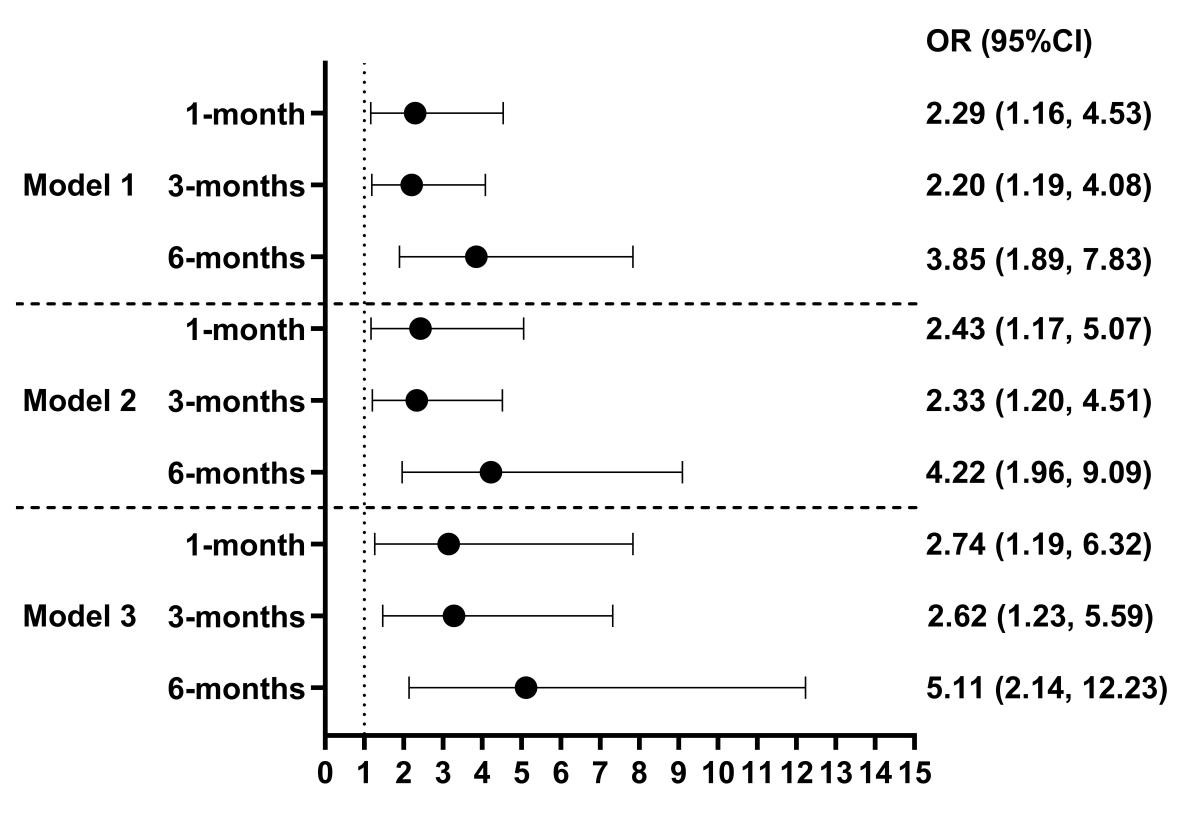


Model 1 was controlled for group and time; Model 2 was also controlled for hospital, age, sex, education, employment status, and marital status; Model 3 was also controlled for smoking, BMI, number of antihypertensive agents, duration of dialysis, IDWG/d, Kt/V*,* and weekly dialysis frequency.
