# Supplementary material for: Efficacy of a Web-Based Home Blood Pressure Monitoring Program in Improving Predialysis Blood Pressure Control Among Patients Undergoing Hemodialysis: Randomized Controlled Trial
Source: JMIR Mhealth Uhealth. 2024 Aug 9;12:e53355. doi: 10.2196/53355 (PMC11350391; doi:10.2196/53355)
Supplement: Multimedia Appendix 2 [file mhealth-v12-e53355-s002.docx]

**Multimedia Appendix 2.** Analysis of the general estimating equations for the effect of the intervention on predialysis blood pressure (n=165).

| Outcomes | Model 1 | | Model 2 | | Model 3 | |
| --- | --- | --- | --- | --- | --- | --- |
|  | β (95% CI) | *P* value | β (95% CI) | *P* value | β (95% CI) | *P* value |
| Pre-dialysis SBP | | |  |  |  |  |
| Group^1)^ | 2.70 (−2.42, 7.82) | .30 | 3.35 (−1.52, 8.21) | .18 | 2.86 (−1.74, 7.45) | .22 |
| Time^2)^ |  |  |  |  |  |  |
| 1 month (T1) | 1.64 (−1.16, 4.44) | .25 | 1.64 (−1.16, 4.44) | .25 | 1.64 (−1.16, 4.44) | .25 |
| 3 months (T2) | 2.29 (−.91, 5.49) | .16 | 2.29 (−.91, 5.49) | .16 | 2.29 (−.91, 5.49) | .16 |
| 6 months (T3) | 2.16 (−1.80, 6.13) | .29 | 2.16 (−1.80, 6.13) | .29 | 2.16 (−1.80, 6.13) | .29 |
| Group^1)^*Time^2)^ |  |  |  |  |  |  |
| 1 month | −1.45 (−6.12, 3.21) | .54 | −1.45 (−6.12, 3.21) | .54 | −1.45 (−6.12, 3.21) | .54 |
| 3 months | −3.18 (−7.55, 1.19) | .15 | −3.18 (−7.55, 1.19) | .15 | −3.18 (−7.55, 1.19) | .15 |
| 6 months | −6.09 (−10.94, −1.24) | .01 | −6.09 (−10.94, −1.24) | .01 | −6.09 (−10.94, −1.24) | .01 |
| Pre-dialysis DBP | | |  |  |  |  |
| Group^1)^ | 1.69 (−1.88, 5.26) | .35 | 1.87 (−1.36, 5.09) | .26 | 1.32 (−1.8, 4.42) | .40 |
| Time^2)^ |  |  |  |  |  |  |
| 1 month (T1) | 1.36 (−.76, 3.48) | .21 | 1.36 (−.76, 3.48) | .21 | 1.36 (−.76, 3.48) | .21 |
| 3 months (T2) | 1.84 (−.002, 3.67) | .05 | 1.84 (−.002, 3.67) | .05 | 1.84 (−.002, 3.67) | .05 |
| 6 months (T3) | 2.77 (.74, 4.80) | .007 | 2.77 (.74, 4.80) | .007 | 2.77 (.74, 4.80) | .007 |
| Group^1)^*Time^2)^ |  |  |  |  |  |  |
| 1 month | −1.07 (−4.89, 2.76) | .59 | −1.07 (−4.89, 2.76) | .59 | −1.07 (−4.89, 2.76) | .59 |
| 3 months | −2.68 (−5.56, .20) | .07 | −2.68 (−5.56, .20) | .07 | −2.68 (−5.56, .20) | .07 |
| 6 months | −4.93 (−7.93, −1.93) | .001 | −4.93 (−7.93, −1.93) | .001 | −4.93 (−7.93, −1.93) | .001 |
| ^1)^ Reference = baseline.  ^2)^ Reference = control group.  Model 1 was controlled for group and time; Model 2 was also controlled for hospital, age, sex, education, employment status, and marital status; Model 3 was additionally controlled for smoking, BMI, number of antihypertensive agents, duration of dialysis, IDWG/d, Kt/V, and frequency of weekly dialysis.  Abbreviations: BP, blood pressure. | | | | | | |
