# Supplementary material for: Efficacy of a Web-Based Home Blood Pressure Monitoring Program in Improving Predialysis Blood Pressure Control Among Patients Undergoing Hemodialysis: Randomized Controlled Trial
Source: JMIR Mhealth Uhealth. 2024 Aug 9;12:e53355. doi: 10.2196/53355 (PMC11350391; doi:10.2196/53355)
Supplement: Checklist 1 [file mhealth-v12-e53355-s003.pdf]

CONSORT-EHEALTH Checklist V1.6.2 Report

Manuscript Number 53355

(based on CONSORT-EHEALTH V1.6), available at [http://tinyurl.com/consort-ehealth-v1-6].

Date completed

7/3/2024 3:15:54

by

tingting chen

Efficacy of Web-based Home Blood Pressure Monitoring Program in Improving Pre-dialysis Blood Pressure Control among Hemodialysis Patients: Randomized Controlled Trial

TITLE

1a-i) Identify the mode of delivery in the title

“Efficacy of Web-based Home Blood Pressure Monitoring Program in Improving Pre-dialysis Blood Pressure Control among Hemodialysis Patients: Randomized Controlled Trial"

1a-ii) Non-web-based components or important co-interventions in title

1a-iii) Primary condition or target group in the title

It's mentioned "hemodialysis patients".

ABSTRACT

1b-i) Key features/functionalities/components of the intervention and comparator in the METHODS section of the ABSTRACT

"multicenter, open-label, randomized controlled trial;1:1 ratio;the Web-based home blood pressure monitoring program; usual care; 6-month period“.

1b-ii) Level of human involvement in the METHODS section of the ABSTRACT

1b-iii) Open vs. closed, web-based (self-assessment) vs. face-to-face assessments in the METHODS section of the ABSTRACT

1b-iv) RESULTS section in abstract must contain use data

1b-v) CONCLUSIONS/DISCUSSION in abstract for negative trials

INTRODUCTION

2a-i) Problem and the type of system/solution

YES, hypertension is a prevalent comorbidity in the hemodialysis population, and blood pressure control is suboptimal. Digital e-intervention models have been demonstrated to be an effective and widely used approach in patients with essential hypertension. Nevertheless, there is a paucity of studies that have applied digital blood pressure intervention models to hemodialysis patients.

2a-ii) Scientific background, rationale: What is known about the (type of) system

There are many advantages of home blood pressure monitoring in hemodialysis patients, including "improving reproducibility , superior diagnostic accuracy , a more intimate nexus with target organ damage, more prognostic information, and serves as a guide for long-term antihypertensive regimens"

Does your paper address CONSORT subitem 2b?

YES, it is mentioned that"our goal was to develop a customized digital BP management program for hypertensive patients undergoing hemodialysis and to evaluate its long-term feasibility, efficacy, and safety in these patients."

METHODS

3a) CONSORT: Description of trial design (such as parallel, factorial) including allocation ratio

"This study was a multicenter, open-label, randomized controlled trial comparing the Web-based home blood pressure monitoring program against usual care for hypertensive patients undergoing hemodialysis" and "A simple randomization procedure was performed by researchers (J. Z and M. Z) using a random number table generated from the list of patients undergoing hemodialysis, at a 1:1 randomization ratio"

3b) CONSORT: Important changes to methods after trial commencement (such as eligibility criteria), with reasons

YES, it's mentioned that "Patients were randomly allocated in a 1:1 ratio to either the Web-based home blood pressure monitoring program as the intervention group or to usual care as the control group over a 6-month period." This program was developed by the research group.

3b-i) Bug fixes, Downtimes, Content Changes

4a) CONSORT: Eligibility criteria for participants

"The inclusion criteria were (a) age between 18 to 80 years; (b) mean pre-dialysis systolic blood pressureof ≥ 140 mmHg and/or diastolic blood pressure of ≥ 90 mmHg over 3 consecutive sessions, and/or use of antihypertensive medication; (c) stage 5 and estimated glomerular filtration rate less than 15 mL/(min·1.73 m2); (d) dialysis initiation period exceeding 3 months; (e) competencies in communication, perception, and learning; and (f) proficiency in smartphone operation, and willingness to participate in the study."

4a-i) Computer / Internet literacy

4a-ii) Open vs. closed, web-based vs. face-to-face assessments:

YES, This study is face-to-face assessments. it 's mentioned that "Participants were enrolled by researchers (Y. C and J. Y). "

4a-iii) Information giving during recruitment

4b) CONSORT: Settings and locations where the data were collected

YES, it's mentioned that "which was performed at dialysis centers of two tertiary hospitals in Guangdong province, China from August 2022 to February 2023. "

4b-i) Report if outcomes were (self-)assessed through online questionnaires

"Sociodemographic and clinical characteristics were collected by a trained investigator using electronic questionnaires and by retrieving clinical data from the charts at the beginning of the study. The pre-dialysis BP values between the two groups were recorded by a trained investigator, and measured during the same period at baseline, and at the 1, 3, and 6-month follow-up. Electronic questionnaires were completed by both groups at the beginning of the study, and at 1-month, 3-month, and 6-month follow-up."

4b-ii) Report how institutional affiliations are displayed

5) CONSORT: Describe the interventions for each group with sufficient details to allow replication, including how and when they were actually administered

5-i) Mention names, credential, affiliations of the developers, sponsors, and owners

5-ii) Describe the history/development process

5-iii) Revisions and updating

5-iv) Quality assurance methods

5-v) Ensure replicability by publishing the source code, and/or providing screenshots/screen-capture video, and/or providing flowcharts of the algorithms used

5-vi) Digital preservation

5-vii) Access

It's mentioned that "To establish a digital conduit for the transmission and recording of BP data, internet-connected sphygmomanometers were utilized. These devices automatically uploaded BP measurement data to the internet cloud for monitoring purposes. The Internet cloud was divided into two ports: the healthcare professional port and the patient port. Both healthcare professionals and patients utilized this cloud on the Internet to collaboratively monitor patients' home blood pressure. ".		
**5-viii) Mode of delivery, features/functionalities/components of the intervention and comparator, and the theoretical framework**		
YES, The implementation of a Web-based home blood pressure monitoring Program for patients in the intervention group. It's mentioned that "The Web-based HBPM program was characterized by a tripartite framework that encompassed intensive one-on-one health education, remote HBPM, and health tweets disseminated through the WeChat platform (Figure. 1)"		
**5-ix) Describe use parameters**		
**5-x) Clarify the level of human involvement**		
**5-xi) Report any prompts/reminders used**		
"They observed abnormal blood pressure instances, including uncontrolled home blood pressure (defined as an average home blood pressure of ≥135/85 mmHg), interdialytic hypotension (defined as a reduction in systolic blood pressure of 20 mm Hg or more, or a decrease in mean arterial pressure by 10 mm Hg, accompanied by symptoms on non-dialysis days), intradialytic hypotension (a decrease in blood pressure accompanied by symptoms in dialysis procedure), and uncontrolled pre-dialysis blood pressure (an average pre-dialysis blood pressure of ≥ 140/90 mmHg). They provided feedback to physicians about abnormal blood pressure and discussed the adjustments to achieve blood pressure management."		
**5-xii) Describe any co-interventions (incl. training/support)**		
YES, it's mentioned that "The Web-based home blood pressure monitoring program was characterized by a tripartite framework that encompassed intensive one-on-one health education, remote home blood pressure monitoring, and health tweets disseminated through the WeChat platform"		
**6a) CONSORT: Completely defined pre-specified primary and secondary outcome measures, including how and when they were assessed**		
"The primary outcomes were the pre-dialysis blood pressure control rate, with targets set at a pre-dialysis blood pressure of less than 140/90 mmHg, pre-dialysis systolic blood pressure and diastolic blood pressure. Secondary outcomes included patient knowledge, perception, and adherence to home blood pressure monitoring."		
**6a-i) Online questionnaires: describe if they were validated for online use and apply CHERRIES items to describe how the questionnaires were designed/deployed**		
**6a-ii) Describe whether and how "use" (including intensity of use/dosage) was defined/measured/monitored**		
**6a-iii) Describe whether, how, and when qualitative feedback from participants was obtained**		
**6b) CONSORT: Any changes to trial outcomes after the trial commenced, with reasons**		
YES, it's mentioned that "which was performed at dialysis centers of two tertiary hospitals in Guangdong province, China from August 2022 to February 2023. "		
**7a) CONSORT: How sample size was determined**		
**7a-i) Describe whether and how expected attrition was taken into account when calculating the sample size**		
**7b) CONSORT: When applicable, explanation of any interim analyses and stopping guidelines**		
"The primary outcomes were the pre-dialysis blood pressure control rate, with targets set at a pre-dialysis blood pressure of less than 140/90 mmHg, pre-dialysis systolic blood pressure and diastolic blood pressure. Secondary outcomes included patient knowledge, perception, and adherence to home blood pressure monitoring."		
**8a) CONSORT: Method used to generate the random allocation sequence**		
YES, it's mentioned that "The random allocation sequence was generated according to the dialysis group through a publicly available online tool (https: //www.random.org/sequences/)."		
**8b) CONSORT: Type of randomisation; details of any restriction (such as blocking and block size)**		
YES, it's mentioned that "A simple randomization procedure was performed by researchers (J. Z and M. Z) using a random number table generated from the list of patients undergoing HD, at a 1:1 randomization ratio. Since patient dialysis management was conducted by different groups, each group of 8-10 patients had a relatively fixed dialysis time, dialysis area, and attending nurses. To minimize bias, the allocation of participants into study groups was conducted using a block randomization procedure. The random allocation sequence was generated according to the dialysis group through a publicly available online tool (https://www.random.org/sequences/)."		
**9) CONSORT: Mechanism used to implement the random allocation sequence (such as sequentially numbered containers), describing any steps taken to conceal the sequence until interventions were assigned**		
YES, it's mentioned that "A simple randomization procedure was performed by researchers (J. Z and M. Z) using a random number table generated from the list of patients undergoing HD, at a 1:1 randomization ratio. Since patient dialysis management was conducted by different groups, each group of 8-10 patients had a relatively fixed dialysis time, dialysis area, and attending nurses. To minimize bias, the allocation of participants into study groups was conducted using a block randomization procedure. The random allocation sequence was generated according to the dialysis group through a publicly available online tool (https://www.random.org/sequences/)." and "This study was a multicenter, open-label, randomized controlled trial".		
**10) CONSORT: Who generated the random allocation sequence, who enrolled participants, and who assigned participants to interventions**		
YES, it's that "Participants were enrolled by researchers (Y. C and J. Y)."; "A simple randomization procedure was performed by researchers (J. Z and M. Z) "; "Two researchers (T. C and W. Z) were responsible for providing online counseling and responding to patients at any time." ; "The outcome assessors (Q. P and C. W) for BP were blinded to the intervention assignment."		
**11a) CONSORT: Blinding - If done, who was blinded after assignment to interventions (for example, participants, care providers, those assessing outcomes) and how**		
**11a-i) Specify who was blinded, and who wasn't**		
YES, it's that "This study was a multicenter, open-label, randomized controlled trial" and "The outcome assessors (Q. P and C. W) for BP were blinded to the intervention assignment."		
**11a-ii) Discuss e.g., whether participants knew which intervention was the "intervention of interest" and which one was the "comparator"**		
**11b) CONSORT: If relevant, description of the similarity of interventions**		
Not applicable, this is a ehealth trial.		
**12a) CONSORT: Statistical methods used to compare groups for primary and secondary outcomes**		
YES, it's mentioned that "Sociodemographic and clinical characteristics and study outcomes were described as mean (standard deviation), or n (%). The comparison of sociodemographic and clinical variables and study outcomes between the two groups was conducted using the independent sample t-tests and Chi-square tests. The primary outcomes were analyzed using generalized estimation equations. Model 1 was adjusted for group and time. Model 2 also controlled for hospital, age, sex, education, employment status, and marital status. In Model 3, smoking, BMI, number of antihypertensive agents, duration of dialysis, IDWG/d, Kt/V, and weekly dialysis frequency were additionally controlled."		
**12a-i) Imputation techniques to deal with attrition / missing values**		
YES, it's mentioned that "All analyses used intention-to-treat principles and missing data were input using the last observation carried forward approach."		
**12b) CONSORT: Methods for additional analyses, such as subgroup analyses and adjusted analyses**		
YES, it's mentioned that "The primary outcomes were analyzed using generalized estimation equations. Model 1 was adjusted for group and time. Model 2 also controlled for hospital, age, sex, education, employment status, and marital status. In Model 3, smoking, BMI, number of antihypertensive agents, duration of dialysis, IDWG/d, Kt/V, and weekly dialysis frequency were additionally controlled."		
RESULTS		
**13a) CONSORT: For each group, the numbers of participants who were randomly assigned, received intended treatment, and were analysed for the primary outcome**		
YES, it's mentioned that "During the recruitment periods, a total of 567 patients undergoing hemodialysis were assessed for eligibility. Among them, 165 enrolled patients were randomly assigned to either the Web-based home blood pressure monitoring program group (n = 84) or the usual care group (n = 81) (Figure. 2)."		
**13b) CONSORT: For each group, losses and exclusions after randomisation, together with reasons**		
YES, please see the "figure 2".		

**13b-i) Attrition diagram**		
**14a) CONSORT: Dates defining the periods of recruitment and follow-up**		
YES, this study "was performed at dialysis centers of two tertiary hospitals in Guangdong province, China from August 2022 to February 2023. " with 6-month follow-up.		
**14a-i) Indicate if critical “secular events” fell into the study period**		
**14b) CONSORT: Why the trial ended or was stopped (early)**		
YES, it's mentioned that"Twenty participants prematurely discontinued the study, primarily due to transferring to other dialysis centers, receiving kidney transplantation, declining further interviews, and death. " Please see the "figure 2".		
**15) CONSORT: A table showing baseline demographic and clinical characteristics for each group**		
YES, ”The mean age of participants was 53.7 (14.0) years and 66.06% were men. The mean duration of HD was 57.8 (45.9) months. Glomerulonephritis (42.2%) was the main etiology of renal disease. Of the patients, 84.8% received treatment for their hypertension, while 37.4% were treated with one antihypertensive agent, and 62.6% were given a combination of antihypertensives. Among those who received a combination of antihypertensives, the combination of calcium channel blockers+β-blockers+angiotensin II receptor antagonists was the most common (31.6%), followed by calcium channel blockers+β-blockers antihypertensives (27.8%). Except for education, other characteristics were similar between the two groups (Table 1).“ Please see the "table 1".		
**15-i) Report demographics associated with digital divide issues**		
YES, please see the "table 1".		
**16a) CONSORT: For each group, number of participants (denominator) included in each analysis and whether the analysis was by original assigned groups**		
**16-i) Report multiple “denominators” and provide definitions**		
YES, it's mentioned that "All analyses used intention-to-treat principles." Please see the "figure 2".		
**16-ii) Primary analysis should be intent-to-treat**		
**17a) CONSORT: For each primary and secondary outcome, results for each group, and the estimated effect size and its precision (such as 95% confidence interval)**		
"(OR = 2.29; 95% CI 1.16–4.53; P = .02), T2 (OR = 2.20; 95% CI 1.19–4.08; P = .01), and T3 (OR = 3.85; 95% CI 1.89–7.83; P < .001)" Please see the table 3 and Appendix 1.		
**17a-i) Presentation of process outcomes such as metrics of use and intensity of use**		
**17b) CONSORT: For binary outcomes, presentation of both absolute and relative effect sizes is recommended**		
Not applicable, the generalised estimating equation analysis used in this study was unable to report absolute and relative effect sizes.		
**18) CONSORT: Results of any other analyses performed, including subgroup analyses and adjusted analyses, distinguishing pre-specified from exploratory**		
"In Model 1, controlling for groups and time effects, a significant difference; The odds of pre-dialysis BP control rate increased slightly after additional adjustment for demographic data of the patients in Model 2, while after further adjustment for the clinical characteristics of the patients in Model 3, the odds markedly increased."		
**18-i) Subgroup analysis of comparing only users**		
**19) CONSORT: All important harms or unintended effects in each group**		
YES, it's mentioned that"Throughout the follow-up period, the Web-based HBPM program group reported 11 instances of hypotension in 9 patients, including 10 instances of intradialytic hypotension and 1 instance of interdialytic hypotension. The control group reported 15 instances of hypotension in 14 patients, including 11 instances of intradialytic hypotension and 4 instances of interdialytic hypotension. No hypotension-related adverse events, such as cardiovascular events (e.g., acute heart attack or ischemic stroke) or falls, were observed in either group."		
**19-i) Include privacy breaches, technical problems**		
**19-ii) Include qualitative feedback from participants or observations from staff/researchers**		
DISCUSSION		
**20) CONSORT: Trial limitations, addressing sources of potential bias, imprecision, multiplicity of analyses**		
**20-i) Typical limitations in ehealth trials**		
YES, it's mentioned that "First, due to the nature of the health behavior intervention, blinding the participants was not feasible. Similarly, healthcare professionals could not be blinded as the same individuals enrolled participants and conducted follow-ups. "		
**21) CONSORT: Generalisability (external validity, applicability) of the trial findings**		
**21-i) Generalizability to other populations**		
**21-ii) Discuss if there were elements in the RCT that would be different in a routine application setting**		
**22) CONSORT: Interpretation consistent with results, balancing benefits and harms, and considering other relevant evidence**		
**22-i) Restate study questions and summarize the answers suggested by the data, starting with primary outcomes and process outcomes (use)**		
"The program led to enhancements in pre-dialysis BP control rates, pre-dialysis BP, patient knowledge, perception, and adherence to HBPM, particularly evident at the 6-month follow-up assessment. These findings support the viability, efficacy, and security of implementing the Web-based HBPM program in patients undergoing maintenance HD. "		
**22-ii) Highlight unanswered new questions, suggest future research**		
Other information		
**23) CONSORT: Registration number and name of trial registry**		
"Trial Registration: China Clinical Trial Registration Center ChiCTR2100051535 "		
**24) CONSORT: Where the full trial protocol can be accessed, if available**		
"https://www.chictr.org.cn/showproj.html?proj=133286. "		
**25) CONSORT: Sources of funding and other support (such as supply of drugs), role of funders**		
"This research was supported by the Natural Science Foundation of Guangdong Province, China (Grant No. 2018A030313514)."		
**X26-i) Comment on ethics committee approval**		
**x26-ii) Outline informed consent procedures**		
**X26-iii) Safety and security procedures**		
**X27-i) State the relation of the study team towards the system being evaluated**		
